# Electronic health record-based prediction models for in-hospital adverse drug event diagnosis or prognosis: a systematic review

**DOI:** 10.1093/jamia/ocad014

**Published:** 2023-02-20

**Authors:** Izak A R Yasrebi-de Kom, Dave A Dongelmans, Nicolette F de Keizer, Kitty J Jager, Martijn C Schut, Ameen Abu-Hanna, Joanna E Klopotowska

**Affiliations:** Amsterdam UMC location University of Amsterdam, Department of Medical Informatics, Amsterdam, The Netherlands; Amsterdam Public Health, Amsterdam, The Netherlands; Amsterdam Public Health, Amsterdam, The Netherlands; Amsterdam UMC location University of Amsterdam, Department of Intensive Care Medicine, Amsterdam, The Netherlands; Amsterdam UMC location University of Amsterdam, Department of Medical Informatics, Amsterdam, The Netherlands; Amsterdam Public Health, Amsterdam, The Netherlands; Amsterdam UMC location University of Amsterdam, Department of Medical Informatics, Amsterdam, The Netherlands; Amsterdam Public Health, Amsterdam, The Netherlands; Amsterdam Cardiovascular Sciences, Pulmonary Hypertension & Thrombosis, Amsterdam, The Netherlands; Amsterdam UMC location University of Amsterdam, Department of Medical Informatics, Amsterdam, The Netherlands; Amsterdam Public Health, Amsterdam, The Netherlands; Amsterdam UMC location Vrije Universiteit Amsterdam, Department of Clinical Chemistry, Amsterdam, The Netherlands; Amsterdam UMC location University of Amsterdam, Department of Medical Informatics, Amsterdam, The Netherlands; Amsterdam Public Health, Amsterdam, The Netherlands; Amsterdam UMC location University of Amsterdam, Department of Medical Informatics, Amsterdam, The Netherlands; Amsterdam Public Health, Amsterdam, The Netherlands

**Keywords:** adverse drug events, prediction models, electronic health records, hospitals, machine learning

## Abstract

**Objective:**

We conducted a systematic review to characterize and critically appraise developed prediction models based on structured electronic health record (EHR) data for adverse drug event (ADE) diagnosis and prognosis in adult hospitalized patients.

**Materials and Methods:**

We searched the Embase and Medline databases (from January 1, 1999, to July 4, 2022) for articles utilizing structured EHR data to develop ADE prediction models for adult inpatients. For our systematic evidence synthesis and critical appraisal, we applied the Checklist for Critical Appraisal and Data Extraction for Systematic Reviews of Prediction Modelling Studies (CHARMS).

**Results:**

Twenty-five articles were included. Studies often did not report crucial information such as patient characteristics or the method for handling missing data. In addition, studies frequently applied inappropriate methods, such as univariable screening for predictor selection. Furthermore, the majority of the studies utilized ADE labels that only described an adverse symptom while not assessing causality or utilizing a causal model. None of the models were externally validated.

**Conclusions:**

Several challenges should be addressed before the models can be widely implemented, including the adherence to reporting standards and the adoption of best practice methods for model development and validation. In addition, we propose a reorientation of the ADE prediction modeling domain to include causality as a fundamental challenge that needs to be addressed in future studies, either through acquiring ADE labels via formal causality assessments or the usage of adverse event labels in combination with causal prediction modeling.

## INTRODUCTION

Adverse drug events (ADEs) in hospitalized patients are common, often preventable, and associated with substantial patient harm.^1^ ADEs have been associated with a significantly prolonged length of stay, increased economic burden, and an almost 2-fold increased risk of death.^2^ Having tools for ADE prediction in hospitalized patients would aid clinicians to recognize or prevent ADEs in a timely manner at the patient level.^3^ Also, insights obtained by such tools on why and when ADEs occur during hospitalization could be used to implement targeted medication safety interventions at the hospital level.^4^ Prediction models, both diagnostic and prognostic, are being increasingly used in the healthcare domain. Prediction models are developed to aid healthcare providers in estimating the probability that a disease or condition is present (diagnosis) or that an event will occur in the future (prognosis). Such models can be applied to inform clinicians and help in decision making.^5^ In the context of ADEs, the diagnostic ADE prediction models could pinpoint patients experiencing an ADE and be used to guide changes in pharmacotherapy. At hospital level, insight into the number and type of ADEs could be used to develop more targeted quality of care interventions. The prognostic ADE prediction models could pinpoint patients at high risk of a future ADE and also be used to guide prescribing decisions to lower the ADE risk.

Previous research has investigated the potential of utilizing prospectively collected data (eg, data from cohort studies, nested case-control, or case-cohort studies) for developing ADE prediction models for inpatients.^6^ Prospectively collected data offer several advantages, including the ability to optimally measure the predictors and the outcome and the inherent blinding of predictor assessment to the outcome occurrence. However, prospective data collection is often costly and limited in the number of patients and predictors.^7^^,^^8^

The implementation of electronic health record (EHR) systems opened the opportunity to reuse data in these systems for ADE diagnosis and prognosis.^9^^,^^10^ Leveraging EHR data for this purpose has several advantages. First, the data are routinely collected and readily available. Second, EHR data can be scanned automatically for (potential) ADEs using computerized algorithms. This presents an attractive alternative to the laborious manual patient chart reviews, which is the current gold standard.^11^ Third, computerized ADE surveillance systems have shown to detect 10 times as many ADEs in comparison to voluntary reporting of ADEs by clinicians (using, eg, local incident reporting systems), a method most hospitals currently use to monitor medication safety.^9^ However, despite the promise that computerized approaches that leverage EHR data hold for identifying (future) ADEs, there are several challenges, such as limited data quality, suboptimal predictive performance, and lack of external validation.^12^^,^^13^ External validation means the validation of the developed model(s) using a separate dataset in which the patients may structurally differ from the patients in the development dataset (patients may for example be from a different geographic region).^5^

There is a lack of detailed insights in the potential of EHR-based prediction models to improve ADE diagnosis and/or prognosis in hospitalized patients. In a recent scoping review of key use cases for artificial intelligence to reduce the frequency of ADEs, promising machine learning models were discussed.^14^ However, no detailed overview and critical appraisal of the developed models were provided. We therefore conducted a systematic review to identify and critically appraise existing EHR-based ADE prediction models. We focused on models which reused structured EHR data (eg, medication administrations, diagnosis codes, laboratory findings), since these data are easier to leverage and require substantially less preprocessing effort in comparison to unstructured data (eg, clinical notes, discharge summaries). Specifically, our primary aim was to provide a systematic overview of properties of the developed ADE prediction models and utilized structured EHR data. Our secondary aim was to identify potential areas for improvement in model development, validation and reporting by critically appraising the included studies. To place our findings into context and identify challenges that are unique to the ADE domain, we compared our findings to that of systematic reviews on prediction modeling in various other medical disciplines published in the past 5 years in peer-reviewed journals by experts on prediction modeling in medicine.^15–26^ These experts were coauthors of an explanatory paper about The Transparent Reporting of a multivariable prediction model for Individual Prognosis Or Diagnosis (TRIPOD) Guideline.^5^ The results of our review are useful for clinical scientists, computer scientists, and healthcare providers working on ADE prediction models based on routinely collected structured EHR data in the adult inpatient setting.

## MATERIALS AND METHODS

### Protocol and registration

This systematic review is reported according to the Preferred Reporting Items for Systematic reviews and Meta-Analyses (PRISMA) Guidelines.^27^ The review protocol was registered as a systematic review at PROSPERO under registration number CRD42020178777 (https://www.crd.york.ac.uk/prospero/display_record.php?ID=CRD42020178777). For data extraction and critical appraisal, the Checklist for Critical Appraisal and Data Extraction for Systematic Reviews of Prediction Modelling Studies (CHARMS) was applied.^7^

### Search strategy

We obtained relevant citations from the Medline and Embase databases using a search strategy consisting of a combination of medical subject headings (MeSH) and keywords related to ADEs, models or algorithms and EHR databases. We included results with a publication date starting from January 1, 1999, written in English. We chose this starting date as we expected that most EHR systems in hospitals were implemented in the last 2 decades. The search strategy was run in Ovid on May 1, 2020. The search was updated on July 4, 2022. In addition, the references of in-scope reviews identified through the database searches were screened to identify additional eligible articles. The full search strategy can be found in Supplementary Appendix S1.

### Study selection and inclusion criteria

The articles retrieved were deduplicated using Endnote version X9.3.3. Rayyan review software was used to conduct the screening of the articles.^28^ Two reviewers (IARY-dK and JEK) independently screened titles and abstracts for potentially eligible studies and disagreements were resolved by consensus. We retrieved full-text articles if the title or abstract indicated the development of (a) model(s) for ADE diagnosis or prognosis in adult inpatients using routinely registered structured EHR data. Full-text articles were screened for eligibility by 2 reviewers (IARY-dK and DAD) and disagreements were again resolved by consensus. We selected articles for inclusion if they (1) were a peer reviewed scientific report of original research, (2) were written in the English language, and (3) developed a diagnostic model to detect ADEs or a prognostic model to predict ADE risk. If the patient setting (ie, outpatient versus inpatient) was unclear, we included the study unless the nature of the ADE indicated an outpatient setting (eg, opioid addiction or ADEs due to prolonged medication usage). Studies were excluded if any of the following applied: (1) informal publication types (eg, conference abstracts, letter to the editor, commentaries), as these are usually not peer reviewed, (2) review articles, since we were interested in original studies to conduct a comprehensive assessment of the developed prediction models, (3) the aim of the study was postmarketing surveillance, given that our goal was to identify ADE prediction models to support healthcare providers in daily clinical practice, (4) the study used so-called triggers (eg, a lab value exceeding a certain threshold pointing to toxicity of a drug) to identify potential ADEs, as such approaches do not use prediction models, and (5) the model was not specified or validated (internally or externally), which would preclude any characterization or comparison on model types or model validation. In addition, we excluded articles where the modeling method utilized unstructured data, since the critical appraisal of such methods requires a specific approach.^29^

### Data charting

One reviewer (IARY-dK) extracted the data from the final selection of articles using a predefined charting table based on CHARMS.^7^ The data extraction was validated for a subset of the included articles (*n* = 5, 20%): a second reviewer (DAD) extracted the data for these articles and disagreements between IARY-dK and DAD were resolved by consensus. Changes in the definitions of the extracted data items following from the consensus were also applied in the data extraction of the remaining articles. The CHARMS checklist provides a comprehensive list of items to extract from included studies to allow for the evaluation of risk of bias and applicability associated with the developed model(s). The items describe crucial elements in prediction model development, including the data source, participant inclusion and exclusion criteria, outcome(s), (candidate) predictors, events per variable (EPV; ie, the number of ADE cases per variable in a multivariable model), missing data, performance metrics and the evaluation method. The collection of the items thereby facilitates the evaluation of the included studies’ adherence to current best practices in prediction model development.^7^ In addition to the items in the CHARMS checklist, we recorded whether the included studies reported the usage of the TRIPOD reporting standard. Furthermore, we collected general information regarding model development including the availability of data and model code and the involvement of key stakeholders before and during model development (eg, clinicians and prediction model experts). In addition, as an ADE inherently describes a causal relationship (ie, *a drug caused an adverse event*), we collected information on whether the approaches to retrieve or generate ADE labels (ie, a variable that specifies if an ADE occurred or not) included a causality assessment by clinicians. Lastly, we recorded whether the included studies reported the implementation of the developed model or whether they were cited by follow-up implementation reports or clinical impact studies. To find such follow-up reports, we identified the studies that cited each of the included studies and subsequently inspected each corresponding title, abstract, and full text to ascertain whether it was an implementation or clinical impact study. We did not conduct a meta-analysis of the quantitative results because we expected high heterogeneity of clinical settings, ADEs and associated prediction model performances.

## RESULTS

**Figure 1.**
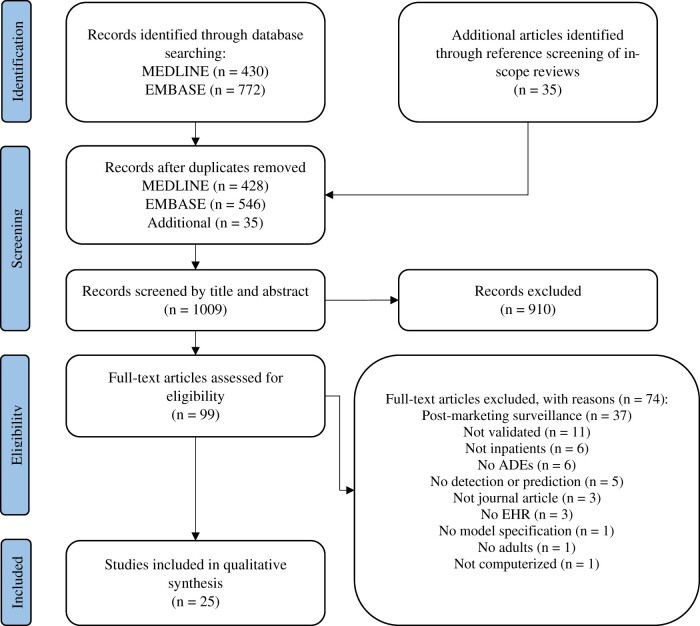
PRISMA flow diagram of the citation search and selection strategy. Adopted from Moher et al.^27^ ADE: adverse drug event; EHR: electronic health record; PRISMA: Preferred Reporting Items for Systematic reviews and Meta-Analyses.

After removing duplicates, 974 citations were identified via database searching. We screened references of in-scope reviews among these citations and identified an additional 35 articles. Of the total 1009 articles, we selected 99 articles based on title and abstract for full-text screening. Of these, 25 articles met our inclusion criteria and were included in the qualitative synthesis.^30–54^ A detailed overview of our selection procedure is shown in a PRISMA flowchart in Figure 1.

The included studies were published from 2012 to 2022, with most studies (68%) published after 2018 (Table 1 and Supplementary Appendix S2).^30^^,^^31^^,^^33–39^^,^^41–45^^,^^49^^,^^50^^,^^54^ Only one of the studies used the TRIPOD reporting guideline to describe the model development and validation.^34^ Twenty studies (80%) reported about prognostic models^31–39^^,^^41–50^^,^^54^ and 5 studies (20%) about diagnostic models.^30^^,^^40^^,^^51–53^ While 17 studies (68%) investigated a single ADE,^31^^,^^33–41^^,^^43–47^^,^^49^^,^^50^ 8 studies (32%) investigated multiple ADEs.^30^^,^^32^^,^^42^^,^^47^^,^^51–54^ Only 1 study (4%) reported the involvement of key stakeholders before model development.^47^ This study was guided by a technical expert panel (including clinical pharmacy specialists, health information technology experts, and researchers in quality measurement and epidemiology) and performed a stakeholder evaluation to identify relevant preventable ADEs before model development. Lastly, none of the included studies reported the implementation of the developed model(s) in clinical practice or was cited by an implementation or clinical impact study (Tables 1 and 2 and Supplementary Appendix S3).

**Table 1.** General description of the included studies

Author (year)	Data source	Data period	Location	Number of centers	Setting	Number of participants/cases
Bagattini et al (2019)^30^	RC	2009–2015	Sweden	1	Inpatient	1597/1047^b^
Choudhury et al (2019)^31^	RC	2012–2017	United States	Multiple^a^	Inpatient and outpatient	86 674/1314
Davis et al (2012)^32^	RC	1960–2012	?	1	?	2294/160^b^
Dong et al (2019)^33^	RC	2000–2017	United States	Multiple^a^	Inpatient	440 000/40 000^c^
Hincapie-Castillo et al (2019)^34^	RC	2012–2013	United States	2	Inpatient	165 847/1672^d^
Imai et al (2019)^35^	RC	2008–2018	Japan	1	?	396/61
Imai et al (2020)^36^	RC	2011–2019	Japan	1	?	1141/179
Jeon et al (2019)^37^	RC	2012–2013	United States	2	Inpatient	62 561/1212
Kim et al (2021)^38^	RC	2016–2018	South Korea	1	Inpatient (ICU)	?^e^
Liang et al (2021)^39^	RC	2017–2019	China	1	?	461/283
Liu et al (2018)^40^	MR	2004–2006	United States	Multiple^a^	?	4350/371
Munoz et al (2019)^41^	RC	2012–2013	United States	2	Inpatient	194 156/262^d^
On et al (2022)^42^	RC	2015–2016	South Korea	19	Inpatient and outpatient	6812/886^f^
Qin et al (2021)^43^	RC	2014–2020	China	1	Inpatient	298/32
Qin et al (2022)^44^	RC	2015–2021	China	1	Inpatient	343/67
Simon et al (2021)^45^	RC	2003–2018	United States	3	Inpatient	35 639/4558
Sun et al (2017)^46^	RC	2000–2017	United States	1	?	4084/80
Winterstein et al (2017)^47^	RC	2012–2013	United States	2	Inpatient	180 875/418^b^
Winterstein et al (2018)^48^	RC	2012–2013	United States	2	Inpatient	60 762/1256^d^
Yang et al (2019)^49^	RC	2011–2017	United States	Multiple^a^	?	17 446/1958
Yuan et al (2021)^50^	RC	2015–2018	United States	18	?	20 579/2102
Zhao et al (2015)^51^	RC	2009^–^2010	Sweden	1	Inpatient and outpatient	4347/113^b^
Zhao et al (2015)^52^	RC	2009–2010	Sweden	1	?	3733/107^g^
Zhao and Henriksson (2016)^53^	RC	2009–2015	Sweden	1	?	9890/217^b^
Zhou et al (2020)^54^	RC	1997–2018	United States	1	Inpatient and outpatient	4309/838^b^

*Note*: ?: unclear based on information provided by the authors.

*Abbreviations*: ADE: adverse drug event; EHR: electronic health record; ICU: intensive care unit; MR: medical registry; RC: retrospective cohort.

a Multiple centers, but exact number unclear.

b Differs per ADE, highest provided.

c Differs across two used databases, highest provided.

d At risk days/days with outcome.

e 811 participants, but multiple cases and controls per participant. Total cases and controls are not provided.

f At risk treatment cycles/treatment cycles with outcome. Differs per ADE, highest provided. Calculated using the reported outcome rates.

g Differs per ADE, highest provided. Calculated using the reported outcome rates.

**Table 2.** Description of the ADEs studied, models, evaluation methods, and best reported performances in the included studies

Author (year)	ADEs studied	Prediction type	Modeling method(s)	Method used for testing model performance	Best performance	Best method
Bagattini et al (2019)^30^	Multiple (15 ADE types)	Diagnosis	RF	IV: 10-fold SCV	0.87 (AUC-ROC)	NA
Choudhury et al (2019)^31^	Antipsychotic induced extra-pyramidal symptoms	Prognosis	SVM, SLP, and LR	IV: TTS (70/30)	0.74 (accuracy)	SLP
Davis et al (2012)^32^	Multiple (3 ADE types)	Prognosis	LUCID	IV: 10-fold SCV	0.44 (AUC-PR)	NA
Dong et al (2019)^33^	Opioid overdose	Prognosis	RF, DT, LR, and NN	IV: TTS (90/10)	0.95 (AUC-ROC)	NN
Hincapie-Castillo et al (2019)^34^	Drug-associated QT prolongation	Prognosis	LR	IV: Bootstrap (100 samples)	0.83 (AUC-ROC)	NA
Imai et al (2019)^35^	Ganciclovir‐induced neutropenia	Prognosis	LR and DT	IV: 10-fold CV for DT	0.85 (accuracy)	Tie
Imai et al (2020)^36^	Vancomycin-induced nephrotoxicity	Prognosis	LR and NN	IV: 10-fold CV for NN	0.86 (accuracy)	NN
Jeon et al (2019)^37^	Drug-induced acute kidney injury	Prognosis	LR	IV: Bootstrap (100 samples)	0.81 (AUC-ROC)	NA
Kim et al (2021)^38^	Drug-induced QT prolongation	Prognosis	LR	IV: TTS	NA	NA
Liang et al (2021)^39^	Pegylated liposomal doxorubicin induced hand foot syndrome	Prognosis	LR	IV: TS	0.79 (AUC-ROC)	NA
Liu et al (2018)^40^	Analgesics induced-cardiovascular disease	Diagnosis	XGBoost, DT, SVM, LR, and GBDT	IV: 10-fold CV	0.92 (AUC-ROC)	XGBoost
Munoz et al (2019)^41^	Medication-associated altered mental status	Prognosis	LR	IV: Bootstrap (100 samples)	0.85 (AUC-ROC)	NA
On et al (2022)^42^	Multiple chemotherapy-induced ADEs (8 ADE types)	Prognosis	LR, DT, and NN	IV: 3-fold CV	0.83 (AUC-ROC)	Tie
Qin et al (2021)^43^	Linezolid-induced anaemia	Prognosis	LR	IV: TS	0.77 (AUC-ROC)	NA
Qin et al (2022)^44^	Linezolid-induced thrombocytopenia	Prognosis	LR	IV: TTS	0.85 (AUC-ROC)	NA
Simon et al (2021)^45^	Drug-induced QT prolongation	Prognosis	NB, LR, RF, and NN	IV: TTS (80/20)	0.71 (AUC-ROC)	NN
Sun et al (2017)^46^	Antineoplastic breast cancer medication induced cardiotoxicity	Prognosis	CPHM	IV: Bootstrapped CV (100 iterations)	0.68 (concordance)	NA
Winterstein et al (2017)^47^	Multiple (16 ADE types)	Prognosis	LR	IV: Bootstrap (100 samples)	0.95 (AUC-ROC)	NA
Winterstein et al (2018)^48^	Drug-induced hypoglycemia	Prognosis	LR	IV: Bootstrap (100 samples)	0.88 (AUC-ROC)	NA
Yang et al (2019)^49^	Cancer therapy-related heart failure	Prognosis	LR, SVM, RF, and GBDT	IV: STTS (80/20)	0.91 (AUC-ROC)	GBDT
Yuan et al (2021)^50^	Contrast-associated acute kidney injury	Prognosis	LR	IV: TTS (temporal)	0.80 (AUC-ROC)	NA
Zhao et al (2015)^51^	Multiple (14 ADE types)	Diagnosis	RF	IV: 5-fold SCV (2 iterations)	0.95 (AUC-ROC)	NA
Zhao et al (2015)^52^	Multiple (27 ADE types)	Diagnosis	CART, DT, SVM, LR, KNN, AB, NB, and RF	IV: 10-fold CV (10 iterations)	0.99 (AUC-ROC)	RF
Zhao and Henriksson (2016)^53^	Multiple (19 ADE types)	Diagnosis	RF	IV: 5-fold SCV (2 iterations)	0.99 (AUC-ROC)	NA
Zhou et al (2020)^54^	Multiple cancer therapy-related ADEs (6 ADE types)	Prognosis	KNN, LR, SVM, RF, and GBDT	IV: TTS (temporal)	0.91 (AUC-ROC)	LR

*Abbreviations*: AB: adaptive boosting; ADE: adverse drug event; AUC-PR: area under the precision recall curve; AUC-ROC: area under the receiver operating characteristic curve; CART: classification and regression trees; CPHM: cox proportional hazard model; CV: cross-validation; DT: decision tree; GBDT: gradient boosting decision tree; IV: internal validation; KNN: k-nearest neighbors; LR: logistic regression; LUCID: latent underlying concept invention on-demand; NA: not applicable; NB: naïve Bayes; NN: neural network; RF: random forests; SCV: stratified crossvalidation; SLP: single layer perceptron; STTS: stratified train/test set; SVM: support vector machine; TTS: train/test set; TS: train set; XGBoost: extreme gradient boosting.

### Characteristics of EHR datasets utilized

Most studies (52%) were conducted in the United States using EHR-based data from US hospitals.^31^^,^^33^^,^^34^^,^^37^^,^^40^^,^^41^^,^^45–50^^,^^54^ The timeframes of the EHR data varied across studies between 2 and 52 years of coverage. Although inclusion and exclusion criteria were described clearly in all except one of the studies,^31^ the patient setting and patient characteristics were missing in 10 (40%)^32^^,^^35^^,^^36^^,^^39^^,^^40^^,^^46^^,^^49^^,^^50^^,^^52^^,^^53^ and 6 (24%)^31–33^^,^^51–53^ studies, respectively (Table 1 and Supplementary Appendix S4).

### ADE labels

The included studies used various approaches to retrieve or generate ADE labels and could broadly be categorized into 3 categories: 5 category I studies using ADEs that were registered by healthcare providers (20%),^30^^,^^33^^,^^51–53^ 3 category II studies using adverse event signals followed by a(n) (formal) ADE causality assessment (12%),^43^^,^^44^^,^^46^ and 15 category III studies using adverse event signals without a(n) (formal) ADE causality assessment (60%).^31^^,^^34–39^^,^^41^^,^^42^^,^^45^^,^^46^^,^^48–50^^,^^54^ Studies in the first category retrieved International Classification of Diseases version 9 or 10 codes (ICD-9 or ICD-10)^55^^,^^56^ that explicitly describe a causal relation between a drug and an adverse symptom (eg, ICD-10 code I95.1: *Hypotension due to drugs*). Studies in the second category generated ADE labels by first retrieving adverse event signals (ie, abnormal clinical findings) and subsequently applying an ADE causality assessment to assess if the detected adverse event signals were actual ADEs. In 2 of these studies, formal ADE causality assessments using the Naranjo probability scale were applied.^43^^,^^44^ Studies in the last category only retrieved adverse event signals without a subsequent (formal) ADE causality assessment. Various abnormal clinical findings were used as adverse event signals, among which: ICD-9 or ICD-10 codes that only described certain symptoms, abnormal lab values, abnormal electrocardiogram (ECG) findings, medication administrations combined with abnormal ECG findings, and medication administrations combined with mental status change assessments or skin and pain assessments. One study utilized methods from both the second and third categories (ICD-9 and ICD-10 adverse event codes and an ADE causality assessment using manual chart review).^46^ For 3 studies (12%), the process of generating or retrieving ADE labels was unclear or reported in a previous publication^32^^,^^40^^,^^47^ (Supplementary Appendix S5). The timeframe of the outcome occurrence (ie, ADE label) was described in 11 studies (44%, Supplementary Appendix S4).^30^^,^^33–38^^,^^41^^,^^45^^,^^47^^,^^48^

### Candidate predictors, sample size, and missing data

The datasets most often included medications (96%),^31–54^ lab measurements (92%),^30–39^^,^^41–48^^,^^50–54^ and patient demographics (84%, Supplementary Appendix S6).^30–37^^,^^39–50^^,^^54^ Only 4 studies (16%) included information describing the definition and measurement method of all candidate predictors.^30^^,^^34^^,^^36^^,^^46^ Fourteen studies (56%) described how predictors were handled during modeling, for example, by reporting the dichotomization of a continuous predictor (Supplementary Appendix S7).^30^^,^^32–37^^,^^41^^,^^49–54^

The studies could roughly be divided into 2 groups when comparing candidate predictors: (1) studies directly utilizing predictors from the EHR data (eg, lab measurements, medication administrations or diagnosis codes, 40%)^30^^,^^32^^,^^33^^,^^38^^,^^40^^,^^45^^,^^49^^,^^51–53^ and (2) studies utilizing EHR data to construct clinical risk factors for an ADE of interest (60%).^31^^,^^34–37^^,^^39^^,^^41–44^^,^^46–48^^,^^50^^,^^54^ For example, Jeon et al^37^ constructed risk factors for drug-induced acute kidney injury, which included a chronic kidney disease (CKD) variable defined as a previous ICD-9 diagnosis for CKD or an estimated historical creatinine clearance below 60 mL/min. Studies that constructed such risk factors often included a limited number of candidate variables (less than 50), while the studies that directly used the candidate predictors from the EHR databases often included many candidate variables (thousands) (Supplementary Appendix S7).

The total number of participants and the number of participants with 1 or more ADEs (ADE cases) in each EHR database showed high variability. For example, Choudhury et al included 86 674 participants and 1314 ADE cases,^31^ while Qin et al^43^ included 298 participants and only 32 ADE cases. The high variability in the number of ADE cases across studies resulted in a high variability in the EPV. Although none of the studies reported the EPV, we calculated it for 12 studies (48%) reporting both the number of ADE cases and the number of variables in the models^34–37^^,^^39^^,^^41–44^^,^^48^^,^^50^^,^^54^; the calculated EPVs ranged between 6 and 262 (Table 1 and Supplementary Appendix S7).

None of the studies reported the number of participants with any missing value or the number of participants with missing values for all predictors. For 4 studies (16%)—however—information about the former fact was implicitly reported as they excluded patients with any missing value.^35^^,^^36^^,^^39^^,^^43^ Sixteen studies (64%) discussed the handling of missing data in the candidate predictors,^30^^,^^34–37^^,^^39–43^^,^^45–48^^,^^50^^,^^54^ most of which used missing data indicators or mean imputation (Supplementary Appendix S7).

### Candidate predictor selection, prediction models, and validation methods

Most studies (76%) discussed the premodeling selection of candidate predictors.^30^^,^^31^^,^^33–39^^,^^41–44^^,^^47^^,^^48^^,^^50–53^ Approaches used by these studies included univariable screening (68%),^30^^,^^34–37^^,^^39^^,^^41–44^^,^^47^^,^^48^^,^^52^ cluster analysis (26%),^34^^,^^37^^,^^41^^,^^47^^,^^48^ and expert opinion (11%).^34^^,^^41^ The most common modeling methods were logistic regression (80%),^31^^,^^33–45^^,^^47–50^^,^^52^^,^^54^ random forests (32%),^30^^,^^33^^,^^45^^,^^49^^,^^51–54^ and decision trees (20%).^33^^,^^35^^,^^40^^,^^42^^,^^52^ Eleven studies (44%) *solely* applied a modeling method commonly regarded as a conventional statistical approach (eg, logistic regression or cox proportional hazard models),^34^^,^^37–39^^,^^41^^,^^43^^,^^44^^,^^46–48^^,^^50^ 4 studies (16%) *solely* applied a machine learning method (eg, random forests),^30^^,^^32^^,^^51^^,^^53^ while the remaining 10 studies (40%) compared multiple methods from the conventional statistical approach and machine learning method categories.^31^^,^^33^^,^^35^^,^^36^^,^^40^^,^^42^^,^^45^^,^^49^^,^^52^^,^^54^ Only 1 study reported the development of a causal prediction model (ie, a prediction model in which the drug exposure’s effect on ADE occurrence may be interpreted causally).^46^ Model assumptions were never discussed, except by Yuan et al^50^ (Table 2 and Supplementary Appendix S8).

All studies conducted internal validation, mainly by using (stratified) cross-validation (36%),^30^^,^^32^^,^^35^^,^^36^^,^^40^^,^^42^^,^^51–53^ bootstrapping (24%),^34^^,^^37^^,^^41^^,^^46–48^ or a single random train/test split (20%).^31^^,^^33^^,^^38^^,^^44^^,^^45^ None of the studies conducted external validation of their models. For most studies, the performance measures included the area under the receiver operating characteristic curve (AUC-ROC, 80%),^30^^,^^33^^,^^34^^,^^36^^,^^37^^,^^39–45^^,^^47–54^ while less than half of the studies (44%) provided a model calibration assessment.^35–38^^,^^40^^,^^43–45^^,^^47^^,^^48^^,^^50^ The highest AUC-ROC reported across the studies varied between 0.71 and 0.99.^30^^,^^33^^,^^34^^,^^36^^,^^37^^,^^39–45^^,^^47–54^ The machine learning methods showed better performances compared to the conventional statistical approaches in 7 of the 10 studies (70%) that presented a comparison between the two^31^^,^^33^^,^^36^^,^^40^^,^^45^^,^^49^^,^^52^ (Table 2 and Supplementary Appendix S8).

## DISCUSSION

ADE prediction models for adult inpatients using structured EHR data have been increasingly studied in the past decade, with most studies published after 2018. Below, we discuss our main findings and recommendations. As prediction models are increasingly developed in many different medical disciplines, we first discuss the similarities between our findings and that of twelve systematic reviews that investigated prediction model development in various other medical disciplines (eg, psychiatry, oncology, neurology).^15–26^ In addition, we discuss and provide suggestions for the most prominent challenge that is specific for the field of ADE prediction modeling: ADE causality.

### General challenges: déjà-vus across disciplines

The identification of a clinically relevant problem is an important part of prediction model development, regardless of the medical discipline. Medical experts are indispensable in this process.^25^^,^^57^^,^^58^ Involving them, along with prediction model experts and implementation experts, serves to identify clinically relevant problems that may be addressed using implementable valid prediction models. Reporting on the process and the involved key stakeholders is important as it increases the support across the different disciplines. Only one of the included studies in our systematic review reported on the involved key stakeholders and identification of a clinically relevant problem prior to model development.^47^ The latter study’s report of this process may serve as an example for future studies.

The included studies in our systematic review often failed to describe crucial elements in the model development process. Examples include a description of the patient setting (ie, intensive care unit), characteristics of the included patients, definitions and measurement methods of predictors, missing data patterns, and how missing data were handled. Failure to report such elements may increase the risk of invalid models and hinders the models’ assessment and reproducibility. Furthermore, without proper specification of the utilized databases and model development and evaluation strategies, claims that machine learning approaches perform better in comparison to conventional statistical approaches cannot be assessed.^59^^,^^60^ Unfortunately, these seem to be a rather common problems across medical disciplines and adherence to reporting standards such as TRIPOD has been recommended repeatedly.^15^^,^^17^^,^^20^^,^^22–24^ The risk of bias and potential usefulness of prediction models can only be adequately assessed if information on all aspects of a prediction model are fully and clearly reported.^5^

Some of the included studies developed models using datasets that contained few patients with an ADE.^30^^,^^52^^,^^53^ This issue has also been identified in other disciplines.^19–21^^,^^23^^,^^25^^,^^26^ Low case counts increase the risk of overfitting, which is characterized by a high performance on a development dataset, but a lower performance on a validation dataset. Although somewhat controversial, an EPV of 10 or more is generally recommended as a rule of thumb for binary outcomes (ie, at least 10 outcome cases per variable), which might be achieved by restricting the number of variables in low case count settings.^7^^,^^61^ Another potential solution may be to increase the dataset size through multicenter collaborations and thereby increase case counts.^12^^,^^25^

Another common and persistent problem is the selection of predictors prior to multivariable modeling based on the univariable association with the outcome,^26^ which was also often applied in the studies included in our report. This method is at risk of predictor selection bias: univariable associations with the outcome may be large but spurious, and the inclusion of such predictors can increase the risk of overfitting and overoptimistic model performance estimates, especially in small datasets.^7^ In regression-based multivariable modeling, multivariable selection techniques during modeling may be used. Although there is no clear optimal approach, some guidelines are provided in existing literature. If sufficient prior knowledge is available on the known predictors of the outcome, one may fit a “full model” that includes all these identified relevant predictors (and no subsequent selection is performed). If existing knowledge is not sufficiently available, backward elimination could be performed instead. Forward selection is generally not recommended as it may lead to overfitting.^7^^,^^62^ Comprehensive guidelines for variable selection in common machine learning algorithms (eg, random forests) are currently lacking. Although such algorithms often (inherently) include variable selection, considerable improvements in performance and parsimony may be achieved through the optimization if this process.^63^ Note that the above variable selection guidelines apply to *prediction models*. For *causal prediction models*, a different approach is needed that addresses confounding bias.^64^^,^^65^

The most widely reported challenge across the inspected reviews was the need for external validation.^16^^,^^19–21^^,^^23–26^ External validation provides the most reliable way to assess model performance in clinical practice, especially across different clinical settings.^5^^,^^66^ None of the included studies conducted external validation and we thus underline the importance of this challenge. Although 2 of the included studies attempted to conduct temporal external validation,^50^^,^^54^ this was done by temporal splitting of 1 single dataset with a continuous timeframe. This is regarded as an intermediate between internal and external validation.^5^

Lastly, clinical implementation was not reported in the included studies or in subsequent follow-up research that cited these included studies, nor any empirical investigations as to how this should be done or what the impact might be. Such findings are similar to that in other disciplines,^16^^,^^18^^,^^23^^,^^25^ and we therefore rearticulate the need for clinical implementation and impact studies.

### ADE prediction models: think causality!

A crucial part in the development of ADE prediction models is the acquisition of accurate ADE labels: which patients developed an ADE, and which did not? ADE labels could be regarded as a “special” type of labels as they specify the presence or absence of a causal relationship in an individual patient: *a drug caused an adverse event.* This differs from other outcome labels—such as disease recurrence or mortality—which do not specify causal relationships in individual patients.

We found that the included studies could be grouped into 3 categories: category I studies using ADEs that were registered by healthcare providers, category II studies using adverse event signals followed by a(n) (formal) ADE causality assessment, and category III studies using adverse event signals only.

The usage of category I ADE labels is problematic because of 4 reasons. First, in an often busy and hectic medical practice, the causality between adverse events and drug exposure is rarely formally assessed, which may result in many false positive ADE labels. A recent study by Wasylewicz et al^67^ showed that of 326 ADEs reported in EHRs by the physicians, only 5% was assessed as a probable ADE and none as a definite ADE according to formal ADE causality assessments. A formal ADE causality assessment is the best practice and encompasses a judgment by an independent team of medical experts of the qualitative probability of a causal relationship between a drug exposure and the adverse event.^11^ Examples include the Naranjo probability scale^68^ and the World Health Organization Collaborating Center for International Drug Monitoring, the Uppsala Monitoring Center (WHO-UMC) criteria.^69^ Depending on how many criteria are satisfied, a causal relationship between the drug exposure and the adverse event is deemed nearly certain, probable, possible or unlikely by the involved expert reviewers. Second, clinicians often fail to recognize a true ADE symptom as drug related. Previous research showed that 20–50% of ADEs in hospitalized patients are not recognized by the medical team.^70–72^ Especially the recognition of ADEs where the drug is involved in a multifactorial pathological condition is problematic.^72^ Third, even if recognized, Kuklik et al^73^ showed that only 1 in 8 ADEs occurring in their sample of inpatients were reported as such by ICD-10 codes in the EHR. Fourth, ICD-9/10 codes are often used for billing purposes and their quality varies according to the experience and expertise of coders. This may have consequences for their validity.^51^^,^^52^ Yet, none of the studies that utilized category I ADE labels reported on the validation of ICD-9/10 codes. Considering the above, an ADE prediction model that is optimized using category I ADE labels may have a low positive predictive value (PPV) and lack appropriate sensitivity.

The usage of category II labels may suffer less from low sensitivity since adverse event signals such as abnormal laboratory findings or abnormal ECG findings may be easily identifiable. Provided that the subsequent causality assessments are formal, this approach may also improve the PPV. However, formal ADE causality assessments are very time consuming.^74^ As hundreds or even thousands of samples might be required to attain appropriate model performance,^75^ this approach may be infeasible.

The majority of the included studies used category III ADE labels. This approach identifies adverse event signals only and does not assess causality. The collection of patients that show adverse event signals will also include (many) patients in which the drug was not the cause of the adverse event and thus confer a low PPV. Importantly, all but one of the studies in this category developed prediction models to provide a diagnosis or prognosis of adverse events that could potentially be ADEs. Such prediction models do not provide an estimation of the increase in risk of the adverse event *due to a drug* for an individual patient; after all, correlation does not imply causation.^76^^,^^77^ Consequently, such a prediction model that predicts adverse events may not be very useful for the diagnosis or prognosis of *ADEs* as it cannot be interpreted causally.

When utilizing category III ADE labels, causal interpretations for individual patients are allowed if the prediction model is a *causal prediction model*, that is, if it sufficiently addresses bias (eg, confounding) and sufficiently captures the functional relationship between the drug and the adverse event (eg, treatment effect heterogeneity).^78^ Only one of the included studies in category III reported the development of a causal prediction model.^46^ A previous report recommended *not* to use category III ADE labels.^79^ However, we argue that—when applied in a *causal prediction modeling framework*—category III ADE labels could serve as rapidly accessible indicators of actual ADEs. Moreover, this approach does not require formal causality assessments prior to fitting the models (as is the case for category II). To optimally assess model performance, a random sample of the individual cases should preferably be presented for a formal causality assessment, which would require a much smaller time investment.

**Figure 2.**
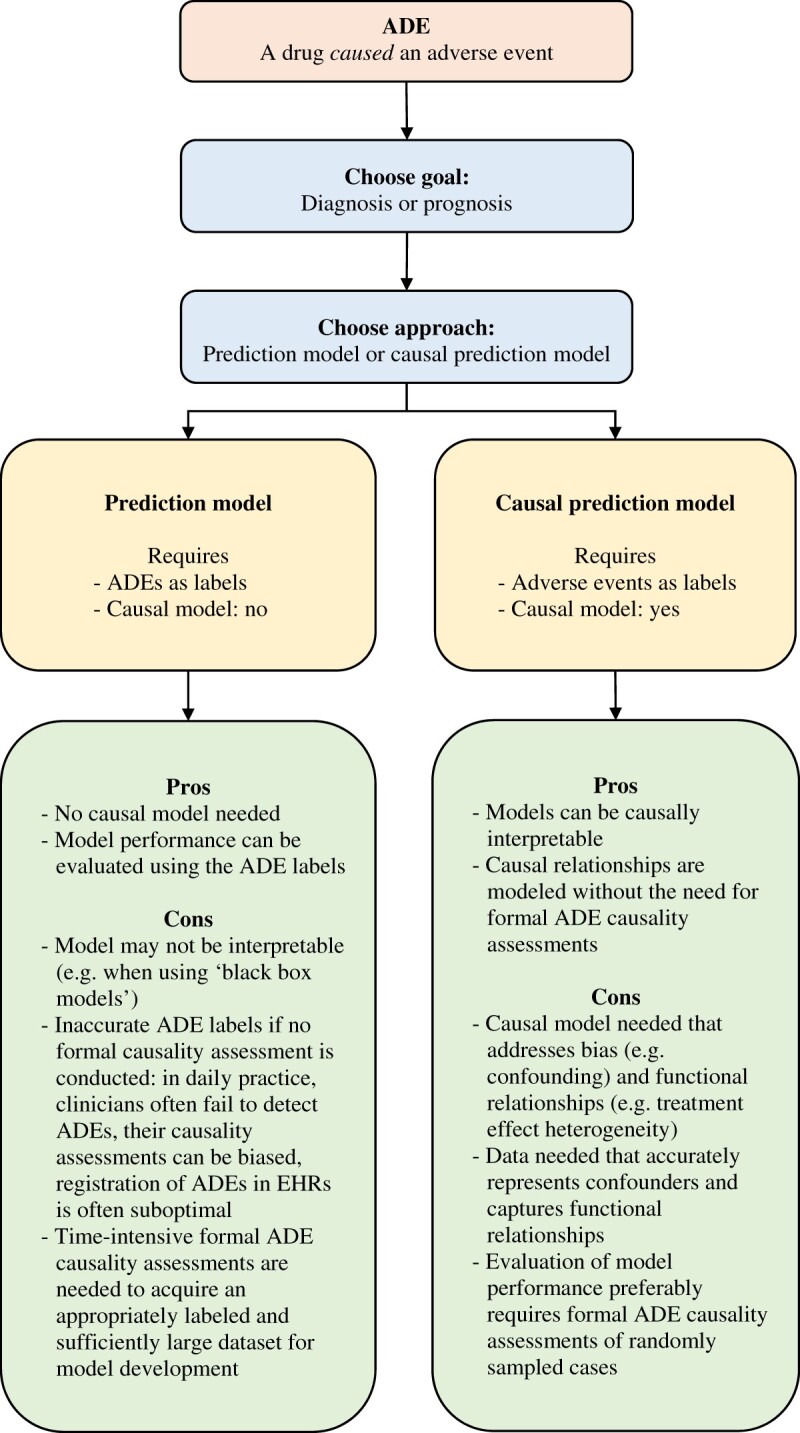
Two approaches to develop ADE prediction models for diagnosis or prognosis. ADE: adverse drug event; EHR: electronic health records.

The 3 categories of utilized ADE labels coincide with 2 ADE prediction model approaches for diagnosis or prognosis. The first approach develops a *prediction model* with category I or II ADE labels, while the second approach develops a *causal prediction model* with category III ADE labels (Figure 2). We propose that causality is a fundamental challenge in ADE prediction modeling and that future studies should explicitly describe how they dealt with this challenge, either through the usage of ADE labels that are the result of formal causality assessments or the usage of adverse event signals in combination with causal prediction modeling.

### Strengths and limitations

The strengths of this review include the use of the CHARMS checklist (a systematic review framework specific for prediction models) for an extensive and systematic critical appraisal of the included studies, a comprehensive search in the Embase and Medline databases and the screening of a large volume of references capturing over 2 decades of research. However, this systematic review also has several limitations. First, we limited the scope of our review to a systematic characterization and critical appraisal of the included studies using the CHARMS checklist to identify potential areas for improvement. Therefore, we did not conduct a formal risk of bias assessment of the developed models using the Prediction model Risk Of Bias ASsessment Tool (PROBAST).^80^ However, most of the PROBAST items are also covered by the CHARMS checklist, albeit without formal judgments on risk of bias. In our discussion, we do however provide our general assessment of these items which fits better with the scope of this review. Second, we restricted our literature search to a period starting from 1999. While none of the included articles were published before 2012, we cannot exclude the possibility that we missed relevant studies from before 1999. However, because EHR implementations were predominantly initiated in the past 2 decades, we deem the risk of having missed such relevant studies as low. Third, we did not conduct a meta-analysis of the quantitative results. We included all ADEs in this systematic review and most studies investigated different ADEs. A meta-analysis would thus have provided limited insights.

## CONCLUSION

Although the development of ADE prediction models for adult inpatients using structured EHR data is increasingly studied, several important challenges should be addressed before the models can be widely implemented. These challenges include the adherence to reporting standards and the usage of model development and validation methodologies that are more in line with current best practices. Importantly, we additionally propose a reorientation of the ADE prediction modeling domain to consider causality as a fundamental challenge that needs to be addressed, either through the usage of ADE labels that are the result of formal causality assessments or the usage of adverse event labels in combination with causal prediction modeling. Addressing these challenges could improve the clinical validity and applicability of ADE prediction models, with promising outlooks for the improvement of medication safety during hospital stay.

## Supplementary Material

ocad014_Supplementary_Data

## Data Availability

The data underlying this article are available in the article and in its online supplementary material.
