# Supplementary material for: Electronic health record-based prediction models for in-hospital adverse drug event diagnosis or prognosis: a systematic review
Source: J Am Med Inform Assoc. 2023 Feb 20;30(5):978–88. doi: 10.1093/jamia/ocad014 (PMC10114128; doi:10.1093/jamia/ocad014)
Supplement: ocad014_Supplementary_Data [file ocad014_supplementary_data.pdf]

Supplementary material

Electronic health record-based prediction models for in-hospital adverse drug event diagnosis or prognosis: a systematic review

Izak A.R. Yasrebi-de Kom[1,2]*, Dave A. Dongelmans[2,3], Nicolette F. de Keizer[1,2], Kitty J. Jager[1,2,4], Martijn C. Schut[1,2,5], Ameen Abu-Hanna[1,2], Joanna E. Klopotowska[1,2]

[1]Amsterdam UMC location University of Amsterdam, Department of Medical Informatics, Meibergdreef 9, Amsterdam, The Netherlands
[2]Amsterdam Public Health, Amsterdam, The Netherlands
[3]Amsterdam UMC location University of Amsterdam, Department of Intensive Care Medicine, Meibergdreef 9, Amsterdam, The Netherlands
[4]Amsterdam Cardiovascular Sciences, Pulmonary hypertension & thrombosis, Amsterdam, The Netherlands
[5]Amsterdam UMC location Vrije Universiteit Amsterdam, Department of Clinical Chemistry, De Boelelaan 1117, Amsterdam, The Netherlands

*Correspondence:
i.a.r.dekom@amsterdamumc.nl
Meibergdreef 9, 1105AZ, Amsterdam, The Netherlands

Supplementary material – Table of contents

Appendix 1. Search strategies ---------- Page 3
Appendix 2. Distribution of study publication years ---------- Page 5
Appendix 3. Reporting on guideline use, code/data availability and stakeholder involvement ---------- Page 6
Appendix 4. Reporting on participants, inclusion/exclusion, treatments and outcomes to be predicted ---------- Page 7
Appendix 5. Description of the ADE labels in the included studies ---------- Page 8
Appendix 6. Description of the EHR database content in the included studies ---------- Page 9
Appendix 7. Reporting on the (candidate) predictors and missing data assessment in the included studies ---------- Page 10
Appendix 8. Reporting on the candidate predictor selection, models and model evaluation in the included studies ---------- Page 11
Supplementary material references ---------- Page 13

Back to table of contents

Appendix 1. Search strategies

Search strategy EMBASE

Terms for "models":
1. model*.ti,ab,kw
2. exp artificial intelligence/
3. artificial adj3 intelligence.ti,ab,kw
4. exp machine learning/
5. (machine adj3 learning).ti,ab,kw
6. neural adj3 network?.ti,ab,kw
7. deep adj3 learning.ti,ab,kw
8. algorithm?.ti,ab,kw
9. support adj1 vector adj1 machine.ti,ab,kw
10. data adj1 mining.ti,ab,kw
11. supervised adj1 Learning.ti,ab,kw
12. unsupervised adj1 Learning.ti,ab,kw
13. 1 or 2 or 3 or 4 or 5 or 6 or 7 or 8 or 9 or 10 or 11 or 12

Terms for "identify":
14. trigger?.ti,ab,kw
15. monitor*.ti,ab,kw
16. score*.ti,ab,kw
17. predict*.ti,ab,kw
18. detect*.ti,ab,kw
19. identif*.ti,ab,kw
20. signal?.ti,ab,kw
21. 14 or 15 or 16 or 17 or 18 or 19 or 20

Terms for "electronic hospital record":
22. (electronic adj3 record?).ti,ab,kw
23. exp electronic health record/
24. 22 or 23

Terms for "ADEs":
25. (adverse adj3 drug adj3 reaction?).ti,ab,kw
26. (adverse adj3 drug adj3 event?).ti,ab,kw
27. ADE?.ti,ab,kw
28. ADR?.ti,ab,kw
29. exp side effect/
30. side adj1 effect?.ti,ab,kw
31. drug adj1 induced.ti,ab,kw
32. drug adj1 related.ti,ab,kw
33. drug-adverse adj1 event.ti,ab,kw
34. exp adverse drug reaction/
35. 25 or 26 or 27 or 28 or 29 or 30 or 31 or 32 or 33 or 34

FINAL EMBASE
36. 13 AND 21 AND 24 AND 35
37. Limit 36 to English language and year=1999-Current

Back to table of contents

Search strategy MEDLINE

Terms for “models”:
1. model*.ti,ab,kf
2. exp artificial intelligence/
3. artificial adj3 intelligence.ti,ab,kf
4. exp machine learning/
5. (machine adj3 learning).ti,ab,kf
6. neural adj3 network?.ti,ab,kf
7. deep adj3 learning.ti,ab,kf
8. algorithm?.ti,ab,kf
9. support adj1 vector adj1 machine.ti,ab,kf
10. data adj1 mining.ti,ab,kf
11. supervised adj1 Learning.ti,ab,kf
12. unsupervised adj1 Learning.ti,ab,kf
13. 1 or 2 or 3 or 4 or 5 or 6 or 7 or 8 or 9 or 10 or 11 or 12

Terms for “identify”:
14. trigger?.ti,ab,kf
15. monitor*.ti,ab,kf
16. score*.ti,ab,kf
17. predict*.ti,ab,kf
18. detect*.ti,ab,kf
19. identif*.ti,ab,kf
20. signal?.ti,ab,kf
21. 14 or 15 or 16 or 17 or 18 or 19 or 20

Terms for “electronic hospital record”:
22. (electronic adj3 record?).ti,ab,kf
23. exp Medical Records Systems, Computerized/
24. 22 or 23

Terms for “ADEs”:
25. (adverse adj3 drug adj3 reaction?).ti,ab,kf
26. (adverse adj3 drug adj3 event?).ti,ab,kf
27. ADE?.ti,ab,kf
28. ADR?.ti,ab,kf
29. side adj1 effect?.ti,ab,kf
30. drug adj1 induced.ti,ab,kf
31. drug adj1 related.ti,ab,kf
32. exp Adverse Drug Reaction Reporting Systems/
33. exp adverse drug reaction/
34. drug-adverse adj1 event.ti,ab,kf
35. 25 or 26 or 27 or 28 or 29 or 30 or 31 or 32 or 33 or 34

FINAL MEDLINE
36. 13 AND 21 AND 24 AND 35
37. Limit 36 to English language and year=1999-Current

Back to table of contents

Appendix 2. Distribution of study publication years

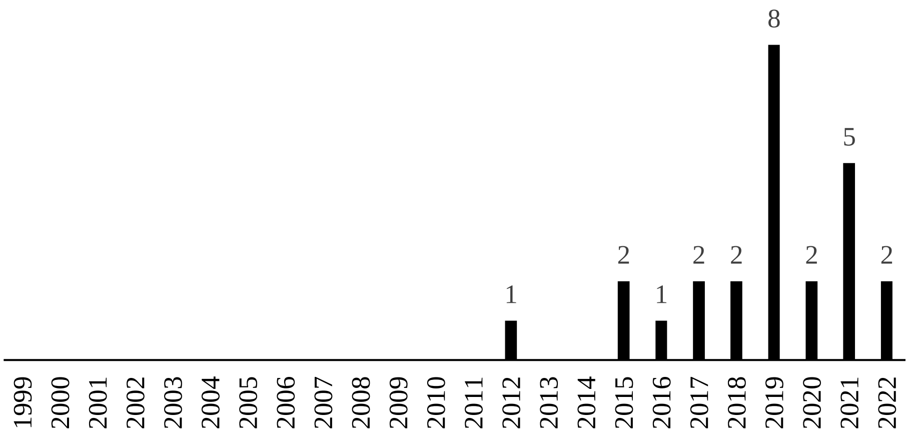

Appendix 2. Distribution of study publication years.

Appendix 3. Reporting on guideline use, code/data availability and stakeholder involvement. ■: yes, ●: no.

Author (year)	Reporting guideline	Code available	Data available	Reported involved stakeholders
Bagattini et al (2019)[1]	●	■	■	●
Choudhury et al (2019)[2]	●	●	●	●
Davis et al (2012)[3]	●	●	●	D
Dong et al (2019)[4]	●	●	●	●
Hincapie-Castillo et al (2019)[5]	■*	●	●	D
Imai et al (2019)[6]	●	●	●	●
Imai et al (2020)[7]	●	●	●	●
Jeon et al (2019)[8]	●	●	●	D
Kim et al (2021)[9]	●	●	●	●
Liang et al (2021)[10]	●	●	■	●
Liu et al (2018)[11]	●	●	●	●
Munoz et al (2019)[12]	●	●	●	D
On et al (2022)[13]	●	●	●	●
Qin et al (2021)[14]	●	●	●	●
Qin et al (2022)[15]	●	●	■	●
Simon et al (2021)[16]	●	●	■	●
Sun et al (2017)[17]	●	●	●	D
Winterstein et al (2017)[18]	●	●	●	B/D
Winterstein et al (2018)[19]	●	●	●	D
Yang et al (2019)[20]	●	●	●	●
Yuan et al (2021)[21]	●	●	■	●
Zhao et al (2015)[22]	●	●	●	●
Zhao et al (2015)[23]	●	●	●	●
Zhao et al (2016)[24]	●	●	●	●
Zhou et al (2020)[25]	●	■	■	●

TRIPOD: Transparent reporting of a multivariable prediction model for individual prognosis or diagnosis, D: during model development, B: before model development.
*TRIPOD[26]

Back to table of contents

Appendix 4. Reporting on participants, inclusion/exclusion, treatments and outcomes to be predicted. ■: yes, ●: no, ? : unclear based on information provided by the authors.

Author (year)	Clear inclusion and exclusion criteria	Participant description	Provided details of treatment received[¶]	Blind outcome assessment	Timeframe of outcome occurrence
Bagattini et al (2019)[1]	■	■ (Table 1)[*]	●	●[‖]	During admission
Choudhury et al (2019)[2]	■	●	●	●[‖]	?
Davis et al (2012)[3]	●	●	●	?	?
Dong et al (2019)[4]	■	●	●	●[‖]	During admission
Hincapie-Castillo et al (2019)[5]	■	■ (Table 1)[†]	■	■[**]	Within the first five admission days
Imai et al (2019)[6]	■	■ (Table 1)	■	■[**]	During the ganciclovir administration period
Imai et al (2020)[7]	■	■ (Table 1)	■	■[**]	During the vancomycin administration period
Jeon et al (2019)[8]	■	■ (Table 2)	■	■[**]	Within the first ten admission days
Kim et al (2021)[9]	■	■ (Table 1)	●	■[**]	During ICU admission
Liang et al (2021)[10]	■	■ (Table 1)	■	●[‖]	?
Liu et al (2018)[11]	■	■[‡]	●	?	?
Munoz et al (2019)[12]	■	■ (Table 2)	■	●[‖]	Within the first five admission days
On et al (2022)[13]	■	■ (Table 1)	■	●[‖]	?
Qin et al (2021)[14]	■	■ (Table 1 and Table 2)	■	●[‖]	?
Qin et al (2022)[15]	■	■ (Table 1, Table 2 and Table 3)	■	●[‖]	?
Simon et al (2021)[16]	■	■ (Table 1)	●	■[**]	During admission
Sun et al (2017)[17]	■	■ (Table 1)	●	●[‖]	?
Winterstein et al (2017)[18]	■	■[§]	●	●[‖]	Within the first five admission days
Winterstein et al (2018)[19]	■	■ (Table 1)	■	■[**]	Within the first five admission days
Yang et al (2019)[20]	■	■ (Table 2)	●	●[‖]	?
Yuan et al (2021)[21]	■	■ (Table 3)	●	■[**]	?
Zhao et al (2015)[22]	■	●	●	●[‖]	?
Zhao et al (2015)[23]	■	●	●	●[‖]	?
Zhao et al (2016)[24]	■	●	●	●[‖]	?
Zhou et al (2020)[25]	■	■ (Table 1)	■	●[‖]	?

ADE: adverse drug event, EHR: electronic health record.
[*] Average age and percentage female per ADE dataset
[†] Additionally referred to other publication
[‡] Referred to Osteoarthritis Initiative data source website: https://oai.epi-ucsf.org/datarelease/default.asp
[§] Referred to previous study[27]
[¶] E.g. duration of therapy or number of concomitant drug exposures
[‖] Unlikely given the outcome assessment method
[**] Automated outcome assessment using EHR

Back to table of contents

Appendix 5. Description of the ADE labels in the included studies. ■: yes, ●: no, ?: unclear based on information provided by the authors.

Author (year)	Clear ADE label definition (data used)	Causality assessed	ADE label category**
Bagattini et al (2019)[1]	■ (ICD-10 codes)	■†	1
Choudhury et al (2019)[2]	■ (ICD-9 and ICD-10 codes)	?‡	3
Davis et al (2012)[3]	●	?	NA
Dong et al (2019)[4]	■ (ICD-9 and ICD-10 codes)	■§	1
Hincapie-Castillo et al (2019)[5]	■ (medication administrations and ECG data)	●	3
Imai et al (2019)[6]	■ (lab values)	●	3
Imai et al (2020)[7]	■ (lab values)	●	3
Jeon et al (2019)[8]	■ (lab values)	●	3
Kim et al (2021)[9]	■ (ECG data)	●	3
Liang et al (2021)[10]	■ (skin and pain assessments)	●	3
Liu et al (2018)[11]	●	?	NA
Munoz et al (2019)[12]	■ (medication administrations mental status change)	●	3
On et al (2022)[13]	■ (manual chart review for ADE symptoms)	●	3
Qin et al (2021)[14]	■ (lab values)	■¶	2
Qin et al (2022)[15]	■ (lab values)	■¶	2
Simon et al (2021)[16]	■ (ECG data)	●	3
Sun et al (2017)[17]	■ (manual chart review, ICD-9 and ICD-10 codes)	■‖	2 and 3
Winterstein et al (2017)[18]	■* (lab values, medication administrations, ECG data, ICD-9 codes, mental status change)	●	3*
Winterstein et al (2018)[19]	■ (lab values)	●	3
Yang et al (2019)[20]	■ (ICD-9 and ICD-10 codes, medication records)	●	3
Yuan et al (2021)[21]	■ (lab values)	●	3
Zhao et al (2015)[22]	■ (ICD-10 codes)	■†	1
Zhao et al (2015)[23]	■ (ICD-10 codes)	■†	1
Zhao et al (2016)[24]	■ (ICD-10 codes)	■†	1
Zhou et al (2020)[25]	■ (ICD-9 and ICD-10 codes)	?‡	3

ADE: adverse drug event, ECG: electrocardiogram, ICD: International Classification of Diseases, EHR: electronic health record, NA: not applicable.
*Reported in a previous publication[27]
†ICD-10 codes explicitly describing an ADE
‡ICD-9 and ICD-10 codes not provided
§ICD-9 and ICD-10 codes explicitly describing an ADE
¶Naranjo causality assessment[28]
‖This study compared two ADE label approaches: (1) Manual chart review followed by causality assessments and (2) ICD-9 and ICD-10 codes describing an adverse event. Likely an unblinded ADE label assessment as manual chart review was applied.
**ADE label categories: 1: ADEs registered by healthcare providers, 2: adverse event signals followed by causality assessments, 3: adverse event signals without causality assessments

Back to table of contents

Appendix 6. Description of the EHR database content in the included studies. ■: yes, ●: no.

Author (year)	Patient demographics	Vital signs	ICD codes	Lab data	Medications	Medical history	Other diagnoses	Physical exams	Nutrition	Time-series utilization*
Bagattini et al (2019)[1]	■	●	■	■	●	●	●	●	●	■
Choudhury et al (2019)[2]	■	●	■	■	■	■	●	●	●	●
Davis et al (2012)[3]	■	■	●	■	■	●	■	●	●	●
Dong et al (2019)[4]	■	●	■	■	■	■	■	●	●	●
Hincapie-Castillo et al (2019)[5]	■	■	●	■	■	■	■	●	●	●
Imai et al (2019)[6]	■	●	●	■	■	■	■	●	●	●
Imai et al (2020)[7]	■	●	●	■	■	●	●	●	●	●
Jeon et al (2019)[8]	■	●	●	■	■	●	■	●	●	●
Kim et al (2021)[9]	●	●	●	■	■	●	●	●	●	●
Liang et al (2021)[10]	■	●	●	■	■	■	■	■	●	●
Liu et al (2018)[11]	■	■	●	●	■	■	●	■	■	●
Munoz et al (2019)[12]	■	■	■	■	■	●	●	■	●	●
On et al (2022)[13]	■	●	■	■	■	■	■	■	●	●
Qin et al (2021)[14]	■	●	●	■	■	■	●	●	●	●
Qin et al (2022)[15]	■	●	●	■	■	■	■	●	●	●
Simon et al (2021)[16]	■	■	■	■	■	■	●	●	●	●
Sun et al (2017)[17]	■	■	■	■	■	●	●	●	●	■
Winterstein et al (2017)[18]	■	●	●	■	■	●	■	●	●	●
Winterstein et al (2018)[19]	■	●	■	■	■	●	●	●	●	●
Yang et al (2019)[20]	■	●	■	●	■	■	●	●	●	●
Yuan et al (2021)[21]	■	●	●	■	■	■	■	●	●	●
Zhao et al (2015)[22]	●	●	■	■	■	●	●	●	●	■
Zhao et al (2015)[23]	●	■	■	■	■	●	●	●	●	■
Zhao et al (2016)[24]	●	■	■	■	■	●	●	●	●	■
Zhou et al (2020)[25]	■	■	■	■	■	●	●	●	●	■

EHR: electronic health record, ICD: International Classification of Diseases.
*Either by using a modeling approach capable of handling time-series data (e.g. cox regression) or a data processing method that preserves time-series dynamics

Back to table of contents

Appendix 7. Reporting on the (candidate) predictors and missing data assessment in the included studies. ■: yes, ●: no, ?: unclear based on information provided by the authors.

Author (year)	Number of candidate predictors	Clear candidate predictor definition and measurement	Clear predictor measurement timing	Predictors assessed blinded for outcome and for each other	Clear handling of predictors in the modeling	Events per variable	Reported number of participants with any missing value	Reported number of participants with missing data for each predictor	Handling of missing data discussed
Bagattini et al (2019)[1]	1877	■	■	■	■	?	●	●	■††
Choudhury et al (2019)[2]	?	●	■	?	●	?	●	●	●
Davis et al (2012)[3]	>9000	●	■	?	■	?	●	●	●
Dong et al (2019)[4]	>8400	●	■	?	■	?	●	●	●
Hincapie-Castillo et al (2019)[5]	34	■	■	?	■	76-104‡	●	●	■‡‡
Imai et al (2019)[6]	34	●	■	?	■	15-20‡	■¶	■¶	■§§
Imai et al (2020)[7]	15	■	■	■	■	22-26‡	■¶	■¶	■§§
Jeon et al (2019)[8]	40	●†	●†	?	■	27-38‡	●	●	■¶¶
Kim et al (2021)[9]	>400	●	■	■	●	?	●	●	●
Liang et al (2021)[10]	26	●	●	?	●	20§	■¶	■¶	■§§
Liu et al (2018)[11]	>300	●	●	?	●	?	●	●	■‖‖
Munoz et al (2019)[12]	>50	●	●	?	■	13§	●	●	■¶¶
On et al (2022)[13]	35	●	■	?	●	13-77‡	●‖	●‖	■
Qin et al (2021)[14]	46	●	●	?	●	6§	■¶	■¶	■§§
Qin et al (2022)[15]	89	●	●	?	●	11§	●	●	●
Simon et al (2021)[16]	6458	●	●	?	●	?	●**	●**	■
Sun et al (2017)[17]	26	■	■	■	●	?	●	●	■***
Winterstein et al (2017)[18]	?	●	■	?	●	?	●	●	■†††
Winterstein et al (2018)[19]	64	●†	●†	?	●	14-23‡	●	●	■‡‡‡
Yang et al (2019)[20]	>7352	●	■	?	■	?	●	●	●
Yuan et al (2021)[21]	20	●	●	?	■	96-262‡	●	●	■§§§
Zhao et al (2015)[22]	≈12230	●	■	?	■	?	●	●	●
Zhao et al (2015)[23]	8262*	●	●	?	■	?	●	●	●
Zhao et al (2016)[24]	6076*	●	●	?	■	?	●	●	●
Zhou et al (2020)[25]	45	●	●	?	■	8-36‡	●	●	■¶¶¶

ADE: adverse drug event.
*May be less depending on the specific ADE
†No information for candidate predictors that were excluded after univariable screening
‡ Based on reported number of outcomes and variables (number of variables varied across models)
§Based on reported number of outcomes and variables
¶These patients were excluded
‖No missing values in patient demographics, chemotherapy type or cancer type. For the other variables the data was coded as binary, with no history of a variable being coded as zero
**No missing values in continuous variables. For the other variables the data was coded as binary, with no history of a variable being coded as zero
††Compared three missing value handling strategies
‡‡Missing variable indicators as candidate predictors for some variables
§§Patients with missing data were excluded
¶¶Missing variable indicators as candidate predictors for some variables, mean imputation for some other variables (other methods than the mean could not be excluded due to the methodology description)
‖‖Variables with >50% missing removed, some variables imputed (using the hot-deck method or a prior or later observation of a time-series variable)
***Missing variable indicators and mean imputation
†††Missing variable indicators or mean or normal imputation for some variables (not clear which)
‡‡‡Missing data indicator variables and mean imputation (other methods than the mean could not be excluded due to the methodology description)
§§§Patients with missing pre or post creatinine were excluded
¶¶¶Patients with >6 missing variables were excluded, remaining missing values were imputed with the mean

Back to table of contents

Appendix 8. Reporting on the candidate predictor selection, models and model evaluation in the included studies. ■: yes, ●: no, ?: unclear based on information provided by the authors.

Author (year)	Reported a causal prediction model	Discussed modeling assumptions	Candidate predictor selection pre-modeling	Candidate predictor selection during-modeling	Shrinkage of predictor weights	Performance measures	Model adjusted or updated	Comparison of predictors values in development / validation datasets
Bagattini et al (2019)[1]	●	●	Univariable screening (information gain)	Selection in RF algorithm (information gain)	●	AUC-ROC (without CI)	●	●
Choudhury et al (2019)[2]	●	●	Selection using previous literature	?	●	Precision, recall, accuracy (with CI, in graph only)	●	●
Davis et al (2012)[3]	●	●	?	Selection using VISTA and AUC-PR	●	AUC-PR, (without CI)	●	●
Dong et al (2019)[4]	●	●	Selection based on occurrence in patients with the outcome	Selection in RF and DT algorithms (Gini impurity), and LIBLINEAR optimization for LR	■	AUC-ROC, accuracy, precision, recall, F-score (without CI)	●	●
Hincapie-Castillo et al (2019)[5]	●	●	Univariable screening, expert screening and cluster analysis	Full model, backward elimination and reduced backward elimination	●	Optimism corrected AUC-ROC (with CI)	●	●
Imai et al (2019)[6]	●	●	Selection of risk factors from previous literature, univariable screening (p-value, for LR)	Stepwise selection for LR and chi-square selection for DT	●	Accuracy for LR and DT (without CI), HL test for LR	●	●
Imai et al (2020)[7]	●	●	Selection of risk factors from previous literature, univariable screening (p-value)	Stepwise selection for LR	●	Accuracy for LR and NN, AUC-ROC for NN (without CI), HL test for LR	●	●
Jeon et al (2019)[8]	●	●	Univariable screening, measurement error assessment and cluster analysis	Full model, backward elimination, reduced backward elimination and an expert model	●	Optimism corrected AUC-ROC (with CI), HL test	●	●
Kim et al (2021)[9]	●	●	Selected the 300 most frequently prescribed drugs and the 100 most frequently done surgeries	?	●	Calibration plot	●	●
Liang et al (2021)[10]	●	●	Univariable screening (p-value)	●	●	AUC-ROC (with CI), accuracy, sensitivity, specificity (without CI), HL test	●	●
Liu et al (2018)[11]	●	●	?	Selection in XGBoost algorithm (candidate screening using loss reduction), no selection discussed for the other algorithms	■	AUC-ROC, AUC-PR, precision, recall, error rate, (without CI)	●	●
Munoz et al (2019)[12]	●	●	Expert screening, univariable screening, merging of variables with low prevalence and similar causal pathways, cluster analysis	Full model, backward elimination and reduced backward elimination	●	Optimism corrected AUC-ROC (with CI)	●	●

Back to table of contents

On et al (2022)[13]	●	●	Selection of risk factors from previous literature, univariable screening (p-value)	Stepwise selection for LR. For the NN model the features in the final LR and DT models were used	●	AUC-ROC, accuracy (without CI)	●	●
Qin et al (2021)[14]	●	●	Univariable screening (p-value)	Forward selection	●	AUC-ROC (with CI), sensitivity, specificity (without CI), HL test	●	●
Qin et al (2022)[15]	●	●	Univariable screening (p-value)	●	●	AUC-ROC (with CI), HL test	●	●
Simon et al (2021)[16]	●	●	?	?	■	AUC-ROC, F-score (without CI), calibration curves	■	●
Sun et al (2017)[17]	■	●	?	?	●	Concordance (with CI, in graph only)	●	●
Winterstein et al (2017)[18]	●	●	Univariable screening, measurement error assessment and cluster analysis	Full model, an expert model, backward elimination and reduced backward elimination	●	AUC-ROC (without CI), HL test	●	●
Winterstein et al (2018)[19]	●	●	Univariable screening and cluster analysis	Full model, an expert model, backward elimination and reduced backward elimination	●	AUC-ROC (with CI), HL test	●	●
Yang et al (2019)[20]	●	●	?	?	■	AUC-ROC, sensitivity, specificity (without CI)	●	●
Yuan et al (2021)[21]	●	■	Selection based on expected availability in EHR	●	●	AUC-ROC, accuracy (without CI), sensitivity, specificity (with CI), HL test	●	■
Zhao et al (2015)[22]	●	●	Sparsity-based selection	Selection in RF algorithm (no further info)	●	AUC-ROC, accuracy, precision, recall, F-score and AUC-PR (without CI)	●	●
Zhao et al (2015)[23]	●	●	Univariable screening (information gain)	Selection in RF algorithm (no further info), no selection discussed for the other algorithms	■	AUC-ROC, accuracy (without CI)	●	●
Zhao et al (2016)[24]	●	●	Assignment of a RF variable importance-based weight to the predictors to subsequent selection probability in RF	Selection in RF algorithm (using weighted probability)	●	AUC-ROC, AUC-PR and accuracy (without CI)	●	●
Zhou et al (2020)[25]	●	●	?	?	■	AUC-ROC, AUC-PR (without CI)	●	●

RF: random forests, LR: logistic regression, LUCID: latent underlying concept invention on-demand, DT: decision tree, NN: neural network, XGBoost: extreme gradient boosting, EHR: electronic health record, AUC-ROC: area under the receiver operating characteristic curve, CI: confidence interval, AUC-PR: area under the precision recall curve, HL: Hosmer–Lemeshow.

Back to table of contents

Supplementary material references

1. Bagattini F, Karlsson I, Rebane J, Papapetrou P. A classification framework for exploiting sparse multi-variate temporal features with application to adverse drug event detection in medical records. *BMC Med. Inform. Decis. Mak.* 2019;**19**(1):7.
2. Predicting adverse drug reactions on distributed health data using federated learning. AMIA Annual symposium proceedings; 2019. American Medical Informatics Association.
3. Davis J, Costa VS, Peissig P, Caldwell M, Berg E, Page D. Demand-Driven Clustering in Relational Domains for Predicting Adverse Drug Events. *Proc Int Conf Mach Learn* 2012;**2012**:1287-94.
4. Machine learning based opioid overdose prediction using electronic health records. AMIA Annual Symposium Proceedings; 2019. American Medical Informatics Association.
5. Hincapie-Castillo JM, Staley B, Henriksen C, Saidi A, Lipori GP, Winterstein AG. Development of a predictive model for drug-associated QT prolongation in the inpatient setting using electronic health record data. *Am. J. Health Syst. Pharm.* 2019;**76**(14):1059-70.
6. Imai S, Yamada T, Kasashi K, Ishiguro N, Kobayashi M, Iseki K. Construction of a flow chart-like risk prediction model of ganciclovir-induced neutropaenia including severity grade: A data mining approach using decision tree. *J. Clin. Pharm. Ther.* 2019;**44**(5):726-34.
7. Imai S, Takekuma Y, Kashiwagi H, et al. Validation of the usefulness of artificial neural networks for risk prediction of adverse drug reactions used for individual patients in clinical practice. *PLoS One* 2020;**15**(7):e0236789.
8. Jeon N, Staley B, Henriksen C, Lipori GP, Winterstein AG. Development and validation of an automated algorithm for identifying patients at higher risk for drug-induced acute kidney injury. *Am. J. Health Syst. Pharm.* 2019;**76**(10):654-66.
9. Kim TY, Choi BJ, Koo Y, Lee S, Yoon D. Development of a Risk Score for QT Prolongation in the Intensive Care Unit Using Time-Series Electrocardiogram Data and Electronic Medical Records. *Healthcare Informatics Research* 2021;**27**(3):182.
10. Liang G, Ma W, Zhao Y, et al. Risk factors for pegylated liposomal doxorubicin-induced moderate to severe hand-foot syndrome in breast cancer patients: assessment of baseline clinical parameters. *BMC Cancer* 2021;**21**(1):1-10.
11. Liu L, Yu Y, Fei Z, et al. An interpretable boosting model to predict side effects of analgesics for osteoarthritis. *BMC Syst. Biol.* 2018;**12**(Suppl 6):105.
12. Munoz MA, Jeon N, Staley B, et al. Predicting medication-associated altered mental status in hospitalized patients: Development and validation of a risk model. *Am. J. Health Syst. Pharm.* 2019;**76**(13):953-63.
13. On J, Park HA, Yoo S. Development of a prediction models for chemotherapy-induced adverse drug reactions: A retrospective observational study using electronic health records. *Eur. J. Oncol. Nurs.* 2022;**56**:102066.
14. Qin Y, Liu Y, Chen Z, Cao M, Shen Y, Ye Y. A risk factor-based predictive model for linezolid-induced anaemia: A 7-year retrospective study. *J. Clin. Pharm. Ther.* 2021.
15. Qin Y, Chen Z, Gao S, Shen Y, Ye Y. Development and validation of a risk prediction model for linezolid-induced thrombocytopenia in elderly patients. *Eur J Hosp Pharm* 2022.
16. Simon ST, Mandair D, Tiwari P, Rosenberg MA. Prediction of Drug-Induced Long QT Syndrome Using Machine Learning Applied to Harmonized Electronic Health Record Data. *J. Cardiovasc. Pharmacol. Ther.* 2021;**26**(4):335-40.
17. Sun D, Simon GJ, Skube S, Blaes AH, Melton GB, Zhang R. Causal phenotyping for susceptibility to cardiotoxicity from antineoplastic breast cancer medications. *AMIA Annual Symposium Proceedings* 2017;**2017**:1655.
18. Winterstein AG, Staley B, Henriksen C, et al. Development and validation of a complexity score to rank hospitalized patients at risk for preventable adverse drug events. *Am. J. Health Syst. Pharm.* 2017;**74**(23):1970-84.
19. Winterstein AG, Jeon N, Staley B, Xu D, Henriksen C, Lipori GP. Development and validation of an automated algorithm for identifying patients at high risk for drug-induced hypoglycemia. *Am. J. Health Syst. Pharm.* 2018;**75**(21):1714-28.
20. Identifying cancer patients at risk for heart failure using machine learning methods. AMIA Annual Symposium Proceedings; 2019. American Medical Informatics Association.
21. Yuan N, Latif K, Botting PG, et al. Refining Safe Contrast Limits for Preventing Acute Kidney Injury After Percutaneous Coronary Intervention. *Journal of the American Heart Association* 2021;**10**(1):e018890.
22. Zhao J, Henriksson A, Kvist M, Asker L, Boström H. Handling temporality of clinical events for drug safety surveillance. *AMIA Annual Symposium Proceedings* 2015;**2015**:1371.
23. Zhao J, Henriksson A, Asker L, Boström H. Predictive modeling of structured electronic health records for adverse drug event detection. *BMC Med. Inform. Decis. Mak.* 2015;**15**(4):1-15.
24. Zhao J, Henriksson A. Learning temporal weights of clinical events using variable importance. *BMC Med. Inform. Decis. Mak.* 2016;**16 Suppl 2**:71.
25. Zhou Y, Hou Y, Hussain M, et al. Machine learning–based risk assessment for Cancer therapy–related cardiac dysfunction in 4300 longitudinal oncology patients. *Journal of the American Heart Association* 2020;**9**(23):e019628.
26. Moons KG, Altman DG, Reitsma JB, et al. Transparent Reporting of a multivariable prediction model for Individual Prognosis or Diagnosis (TRIPOD): explanation and elaboration. *Ann. Intern. Med.* 2015;**162**(1):W1-73.
27. Jeon N, Sorokina M, Henriksen C, Staley B, Lipori GP, Winterstein AG. Measurement of selected preventable adverse drug events in electronic health records: Toward developing a complexity score. *Am. J. Health Syst. Pharm.* 2017;**74**(22):1865-77.
28. Naranjo CA, Busto U, Sellers EM, et al. A method for estimating the probability of adverse drug reactions. *Clin. Pharmacol. Ther.* 1981;**30**(2):239-45.
